# A Randomised Controlled Trial of Local Infiltration Analgesia Versus Femoral Nerve Block for Postoperative Analgesia Following Total Knee Arthroplasty

**DOI:** 10.7759/cureus.10192

**Published:** 2020-09-02

**Authors:** Yang Min Ng, Fiona Martin, Hugh B Waterson, Adam Green, Jeremy Preece, Nerida Robinson, Jon Phillips, Keith S Eyres, Andrew D Toms, James Simpson

**Affiliations:** 1 Anaesthesiology, Royal Devon & Exeter NHS Foundation Trust, Exeter, GBR; 2 Orthopaedic Surgery, Royal Devon & Exeter NHS Foundation Trust, Exeter, GBR; 3 Anaesthesiology, University Hospitals Plymouth NHS Trust, Plymouth, GBR; 4 Anaesthesiology, Northern Devon Healthcare NHS Trust, Barnstaple, GBR; 5 Anaesthesiology, The Canberra Hospital, Canberra, AUS; 6 Orthopaedics, Royal Devon & Exeter NHS Foundation Trust, Exeter, GBR

**Keywords:** total knee arthroplasty, femoral nerve, local anaesthetic

## Abstract

Background

Total knee replacement is often associated with significant postoperative pain. Although the use of a femoral nerve block is well-established, local infiltration analgesia has gained popularity in recent years. We compared single-shot local infiltration analgesia with a single-shot femoral nerve block for patients undergoing primary total knee arthroplasty.

Methods

A total of 194 patients were randomised to receive either local infiltration analgesia (150 ml bupivacaine 0.067% with adrenaline) or a femoral nerve block (20 ml 0.375% levobupivacaine). Both groups received spinal anaesthesia. The primary outcome measure was the total morphine consumption. Secondary outcome measures included: post-operative pain scores, rehabilitation goals, readiness for discharge, and physical, mental, and functional outcomes, including the Oxford Knee Score (OKS).

Results

A total of 69 patients in the local infiltration analgesia group and 79 patients in the femoral nerve block group were analysed. Median total morphine consumption was significantly greater in the local infiltration analgesia group as compared to the femoral nerve block group (54.67 mg vs 45 mg, respectively, p=0.0388). The post-operative OKS at six weeks was slightly more improved for the femoral nerve block group than for local infiltration analgesia (12.5 vs 9 point median improvements for the femoral nerve block and local infiltration analgesia groups, respectively, p=0.0261). There were no statistically significant differences in other secondary outcome measures.

Conclusion

A single-shot femoral nerve block significantly reduces the opioid requirement for primary total knee arthroplasty but is otherwise comparable to single-shot local infiltration analgesia.

## Introduction

Total knee arthroplasty (TKA) is often associated with significant early post-operative pain. There is an inherent conflict between the need to rest the operated limb to limit the pain and the need to move it to avoid stiffness, facilitating a timely recovery [1]. A femoral nerve block (FNB) has long been advocated as an effective part of the post-operative analgesia regime for TKR [2]. In recent years, however, local infiltration analgesia (LIA) has gained popularity, whereby surgeons inject a relatively large volume of a dilute local anaesthetic directly into and around the operative site. LIA has since been embraced at our centre, with potential advantages over other methods, including speed, simplicity, and reduced postoperative weakness.

Multiple studies have compared LIA with other regional techniques, but many of these looked at catheter-based techniques rather than the single-shot technique [3-5]. Our local experience has found that catheter techniques are time-consuming in the theatre and can be difficult to manage in the ward. There are also concerns about the increased risk of post-operative infections, which can be catastrophic [6]. At the start of our study in 2015, few trials directly compared single-shot techniques.

We, therefore, conducted a single-centre, randomised controlled trial of single-shot LIA versus single-shot FNB for postoperative analgesia following TKA.

## Materials and methods

Ethical approval was granted by the South West (Exeter) Research Ethics Service Committee. The trial was conducted at the Royal Devon & Exeter NHS Foundation Trust in the UK and prospectively registered with ClinicalTrials.gov (Identifier: NCT0228892).

We screened and recruited patients undergoing primary TKA from the pre-operative assessment clinic. We excluded patients having TKA for trauma and unicompartmental and bilateral surgery, patients with contraindications to spinal anaesthesia or peripheral nerve blocks, allergy to local anaesthetics or morphine, and problems with communication that would have compromised informed consent. We also excluded patients under active management by chronic pain services, patients with chronic strong opioid use (e.g. morphine, oxycodone, buprenorphine and methadone), or patients taking neuropathic analgesic agents (e.g. gabapentin, pregabalin, or amitriptyline). Pre-operative physical and mental health and functional status were assessed using the Oxford Knee Score (OKS), the EuroQol 5 Dimension Score (EQ-5D-5L), and the Hospital Anxiety and Depression Scale (HADS).

Based on a previous study conducted at our hospital [7], we expected patients in the FNB group to use a median of 44 mg morphine in total over 72 hours. In order to make our results as robust as possible, we set an effect size of 0.4, making a difference in total morphine consumption between the two groups of >40% as clinically significant. With a significance level of <0.05 and power of 80%, we calculated that at least 64 patients per group would be required.

We used block randomisation with numbered, sealed envelopes, which informed clinicians of the relevant protocol shortly before surgery. It was considered neither practical nor safe to blind the healthcare staff directly involved with patient care to the treatment arm of the study. Those collecting, reviewing, and analyzing the data were, however, blinded and patients were not informed of their allocation arm.

All patients received a spinal anaesthetic containing 2.5-3.5 ml 0.5% plain levobupivacaine. Additional sedation or general anaesthesia (including intravenous fentanyl up to 2 μg/kg) was administered at the discretion of the anaesthetist. All patients received the same type of total knee arthroplasty performed with a standard technique without the use of a tourniquet.

Patients in the LIA group received a single shot of 40 ml 0.25% bupivacaine with adrenaline 1:200,000, diluted with 110ml of 0.9% saline, making a total volume of 150ml. This was divided into thirds and administered by the surgeon towards the end of the operation: 50 ml was injected into the posterior capsule before cementing, 50 ml into the medial and lateral capsules, and 50 ml into subcutaneous tissues and in and around the vastus medialis and sartorius muscles.

Patients in the FNB group received a single-shot femoral nerve block before the operation using 20 ml 0.375% levobupivacaine, guided by ultrasound or a peripheral nerve stimulator.

Postoperatively, all patients received morphine patient-controlled analgesia (PCA) pump and regular paracetamol and ibuprofen (unless contraindicated or already on an alternative non-steroidal anti-inflammatory drug). Oral morphine was prescribed for all patients following the discontinuation of their PCA, usually on postoperative day 1.

The primary outcome measure was total morphine consumption during the first 72 hours postoperatively, measured during the following four time periods: 0-12 hours, 12-24 hours, 24-48 hours, and 48-72 hours. This was expressed as the intravenous equivalent dose of morphine (i.e., the total intravenous dose via boluses and the patient’s PCA, plus one-third of any oral morphine administered).

Secondary outcome measures were: postoperative pain scores using the Numerical Rating Scale (NRS); rehabilitation goals (stand and sit out by postoperative day 1, walk to the bathroom by postoperative day 2, walk independently with crutches by postoperative day 4); medical fitness for discharge; Quality of Recovery 40 (QoR-40) questionnaire; Oxford Knee Score (OKS) and EuroQol 5 Dimensions Score (EQ-5D-5L), both collected preoperatively and six weeks postoperatively.

Statistical analysis was undertaken using IBM SPSS Statistics for Windows v20.0 (IBM Corp., Armonk, NY). Shapiro-Wilk was used to test for parametricity. Where parametric data were identified, means and standard deviations were reported. Where non-parametric data were identified, medians and interquartile ranges were reported. For statistical significance, we used the Mann-Whitney U test for median total morphine consumption, OKS, 5Q-5D-5L scores, QoR scores, and readiness for discharge; the student’s unpaired t-test for pain scores, and the chi-squared test for complications. We considered p < 0.05 to be statistically significant.

## Results

**Figure 1:**
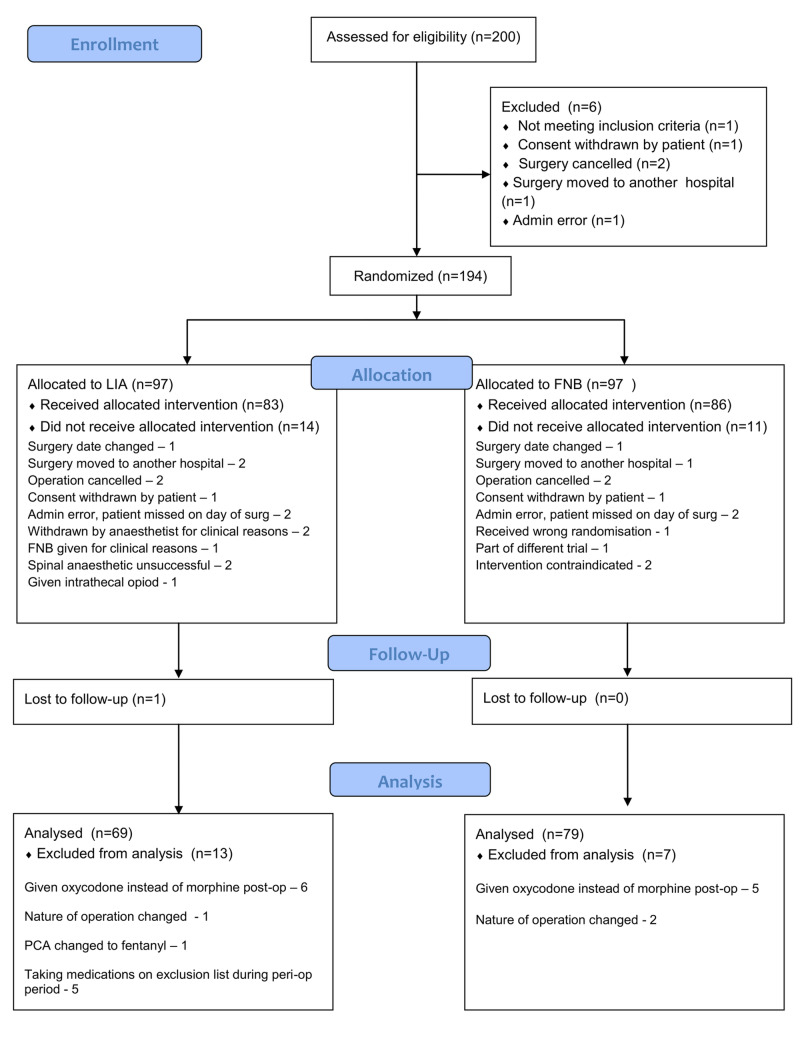
CONSORT diagram of patient recruitment

The study was conducted between March 2015 and July 2018. We recruited a total of 200 patients (Figure 1). Six were excluded and the remaining 194 were randomised. Of these, 25 patients did not receive the allocated intervention so were withdrawn (14 in LIA and 11 in FNB groups, respectively). Of note, in the LIA group, three patients were deemed inappropriate to receive LIA alone and, therefore, FNB was administered.

One patient in the LIA group was lost to follow-up, and 20 patients were excluded from the analysis due to protocol violations (13 in LIA and 7 in FNB groups, respectively). Of these, a total of 12 patients across both groups were excluded, as they were prescribed non-morphine analgesics postoperatively. This included 11 patients who were given oxycodone, and one patient in the LIA group was prescribed fentanyl PCA. The final analysis was performed on an ‘as treated’ basis. Following exclusions, we analysed 148 patients.

Baseline characteristics, including gender, OKS, and EQ-5D-5L scores, were well-matched and comparable in both intervention groups (Table 1) (p>0.05 for all preoperative scores). Ninety-seven point six (97.6%) of patients were Caucasian British. The proportions of both groups having operations in the morning (LIA 35; FNB 35), the afternoon (LIA 42; FNB 50), and postoperative care in each hospital ward were not significantly different. The majority of operations (n=145, 98%) were conducted by two surgeons. Three anaesthetists performed the most (n=62, 41.8%), but 17 anaesthetists were involved in only one operation.

**Table 1:** Baseline measures pre-intervention EQ-5D-5L = 5-level EuroQol 5 Dimension Score; OKS = Oxford Knee Score; HAD = Hospital Anxiety & Depression Scale; SD = Standard Deviation

	LIA (n=69)	FNB(n=79)
Gender, n, M/F	38/31	40/39
Pre-EQ-5D-5L Total Score, mean (SD)	12.48 (3.2)	13.17 (2.66)
Pre-OKS, mean (SD)	39.63 (7.48)	40.17 (6.82)
Pre-HAD Total Score, mean (SD)	8.94 (5.1)	10.63 (6.34)

Primary outcome measure

The median total morphine consumption was significantly greater in the LIA group than in the FNB group (54.67 mg and 45 mg, respectively, p=0.0388, Table 2), a median difference of 10.67 mg.

**Table 2:** Postoperative morphine consumption expressed in mg, intravenous dose equivalent Figures expressed as median values and interquartile range

	LIA (n=69)	FNB (n=79)	p-value
Recovery	0 (0-1)	0 (0-0)	
0-12 hours	19.5 (11-29)	13 (6.8-19.0)	
13-24 hours	4.5 (1-12)	2 (0.5-6)	
25-48 hours	15.67 (6.7-27)	14 (6.8-25)	
49-72 hours	4.67 (0-12)	6.67 (0-13.3)	
Total Morphine consumption	54.67 (28-85)	45 (24.2-62.3)	0.0388

Secondary outcome measures

Pain Scores

**Figure 2:**
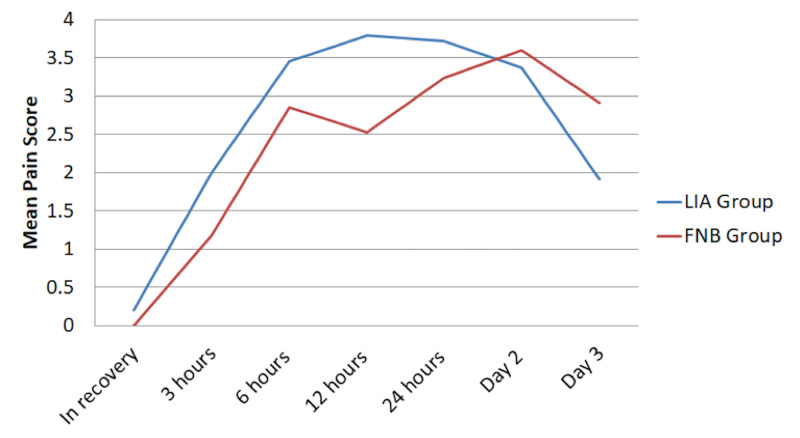
Postoperative pain scores at different time intervals postoperatively scaled from 0-10, with 0 being no pain and 10 being the worst pain ever experienced Figures expressed as mean values.

Mean pain scores were negligible or zero in recovery, peaked at 12 hours (LIA group) and postoperative day 2 (FNB group), before decreasing on postoperative day 3 in both groups (Table 3). A split plot was used to look for repeated-measure and between-groups effects (Figure 2). However, the differences in overall pain score were not statistically significant (p=0.3129).

**Table 3:** Postoperative mean pain scores scaled from 0-10, with 0 being no pain and 10 being the worst pain ever experienced Figures expressed as mean values and 95% confidence interval.

	LIA (n=69)	FNB (n=79)	p-value
Pain score in recovery	0.2 (0-0.452)	0 (0-0)	
Pain score at 3 hours	2 (1.29-2.71)	1.18 (0.675-1.69)	
Pain score at 6 hours	3.46 (2.75-4.17)	2.85 (2.24-3.46)	
Pain score at 12 hours	3.79 (3.79-2.61)	2.53 (1.95-3.11)	
Pain score at 24 hours	3.72 (3.13-4.31)	3.24 (2.7-3.78)	
Pain score at postop Day 2	3.37 (2.73-4.01)	3.6 (3.06-4.14)	
Pain score at postop Day 3	1.91 (1.36-2.46)	2.91 (2.32-3.5)	
Overall pain score in the first 72 hours	2.50 (2.19-2.80)	2.29 (2.03-2.56)	0.3129

Achievement of Rehabilitation Goals

A higher proportion of patients in the FNB group achieved rehabilitation goals at postoperative day 1, but the differences were less apparent after that and did not reach significance at any stage (p=0.145, Table 4).

**Table 4:** Proportion of patients reaching rehabilitation goals, whereby patients are expected to stand and sit out by end of postop day 1, able to walk to the bathroom by end of day 2, and walking independently with crutches by end of day 4

	LIA	FNB	P-value
Postop Day 1	53/69 (76.8%)	68/79 (86.1%)	
Postop Day 2	53/69 (76.8%)	63/79 (79.5%)	
Postop Day 4	62/39 (89.9%)	67/79 (85.1%)	
Ready to discharge (median)	Day 3	Day 4	0.2941

Readiness for Discharge

There was no significant difference in time to discharge between the two groups. The median number of days was 4 (interquartile range 3-5) and 3 (interquartile range 3-4) for the FNB and LIA groups, respectively (p=0.2941). 

Patient Recovery, Satisfaction, and Functional Improvement

Postoperative Quality of Recovery 40 (QoR-40) and 5-level EuroQol 5 Dimension Score (EQ-5D-5L) scores were not significantly different between the groups (Table 5). There was, however, a statistically significant difference in the improvement of the Oxford Knee Score six weeks postoperatively, with a median improvement of 12.5 points in the FNB group vs 9 in the LIA group (p=0.0261).

**Table 5:** Postoperative physical, mental and functional status questionnaire scores QoR-40 = Quality of Recovery 40 Questionnaire; EQ-5D-5L = 5-level EuroQol 5 Dimension Score; OKS = Oxford Knee Score. Figures expressed as median scores and (interquartile range)

	LIA (n=69)	FNB (n=79)	P-value
QoR-40 total score (day 2 postop)	175 (162-181)	176 (154-187)	0.3642
EQ-5D-5L total score (6 weeks postop)	9 (7-10)	9 (6-11)	
Improvement in EQ-5D-5L total score	3	5	0.2749
OKS total score (6 weeks postop)	27 (22-33)	26 (20-35)	
Improvement in OKS score	9	12.5	0.0261

Complications

There was one death in the LIA group. The patient developed multiorgan failure and subsequently died. There was a greater number of major and minor complications in the FNB Group (17/79, 21.52%) than in the LIA group (10/69, 14.49%), but the difference was not statistically significant (Risk Ratio 1.4, 95% CI: 0.69 to 2.86, p=0.2695) (Table 6). The largest percentage of minor complications were wound site infections, and these cannot be attributed to the FNB.

**Table 6:** Major and minor complications Figures expressed as n (%)

LIA (n=69)	FNB (n=79)
Major Complications	Major Complications
Death – 1 (1.4%)	Pneumonia – 1 (1.3%)
	Myocardial infarction – 1 (1.3%)
Minor Complications	Minor Complications
Minor wound site infection – 2 (2.9%)	Minor wound site infection – 7 (8.9%)
Fall – 1 (1.4%)	Fall – 1 (1.3%)
Urinary retention – 2 (2.9%)	Delirium – 2 (2.5%)
Acute renal impairment – 1 (1.4%)	Urinary tract infection – 2 (2.5%)
Other complications – 3 (4.3%)	Cardiac dysrhythmia –1 (1.3%)
	Pain and poor rehabilitation 1 (1.3%)
	Locked knee 1 (1.3%)

## Discussion

Our study demonstrated that patients in the FNB group used significantly less morphine postoperatively than the LIA group. The difference was particularly apparent during the first 24 hours. Whilst there was no significant difference between pain scores, given that there was rescue analgesia immediately available during the early postoperative period in the form of patient-controlled analgesia, we expect that those in more pain simply used more morphine.

We also noted a slightly better improvement in the postoperative Oxford Knee Score for the FNB group. This may indicate that the FNB group had subjectively less pain and better function at six weeks postoperatively when compared to the LIA group. However, the clinical relevance of this small numerical difference is unclear, and the difference between the groups (12.5 points in the FNB group vs 9 in the LIA group) was smaller than the minimal clinically important difference for the Oxford Knee Score [8].

There was one postoperative fall in each group, but for the one in the FNB group, the non-operative leg was at fault. There were no falls relating to weakness from FNB. However, given how catastrophic a fall may be, adductor canal blocks have gained traction in recent years, as they relatively spare quadriceps strength when compared to FNB [9]. Given the elderly and frequently comorbid population presenting for TKA surgery, the other complications highlighted in Table 6 were not considered to be unusual or likely to have been related to the treatment interventions of the study.

The FNB group appeared to achieve readiness for discharge one day later than the LIA group. However, this difference was not statistically significant.

There were several potential limitations to our study. The total masses of bupivacaine and levobupivacaine used were different between the groups (100 mg vs 75 mg for the LIA and FNB groups, respectively). This was because the doses of bupivacaine and levobupivacaine for both interventions were standard at our institution at the time of the study. The LIA mixture was comparable to others in both constituents and volume [10]. Twenty ml of 0.375% levobupivacaine for the FNB was chosen to strike a balance between adequate analgesia and prolonged motor blockade, which may adversely affect mobilisation and rehabilitation. It, therefore, follows that the true differences between FNB and LIA may be even greater than demonstrated by our study.

Other limitations include the number of different anaesthetists involved and that some patients received a general anaesthetic in addition to their spinal anaesthetic. We allowed individual anaesthetists to utilise either ultrasound or nerve stimulator as per their usual practise to administer femoral nerve blockade. There was a need to balance a rigid protocol with real-world flexibility to facilitate service delivery, minimise protocol violations, and complete recruitment within a reasonable timeframe. Our study was thus designed to reflect a reasonable compromise, which was felt unlikely to have a significantly detrimental effect on the validity of our results.

Following the completion of our trial, several studies and meta-analyses have since been published comparing LIA with FNB [11-18]. Our trial supported many of the findings of these studies, including the lack of differences in postoperative pain scores, rates of complication, and length of hospital stay. However, our trial highlighted a significant difference in morphine consumption, with less morphine use in the FNB group. Our trial was also unique in identifying slightly better improvement in long-term functional outcome for patients who have received femoral nerve blockade, although the clinical relevance of this is unclear.

## Conclusions

We conclude that single-shot LIA is comparable to single-shot FNB for primary total knee arthroplasty. However, FNB significantly reduces the opioid requirement. FNB, therefore, remains an important tool and should be considered especially in patients where analgesic strategies may be challenging.
